# Efficacy of Core Training in Swimming Performance and Neuromuscular Parameters of Young Swimmers: A Randomised Control Trial

**DOI:** 10.3390/jcm11113198

**Published:** 2022-06-03

**Authors:** Ahmad Khiyami, Shibili Nuhmani, Royes Joseph, Turki Saeed Abualait, Qassim Muaidi

**Affiliations:** 1 Department of Physical Therapy, College of Applied Medical Sciences, Imam Abdulrahman Bin Faisal University, Dammam 31451, Saudi Arabia; 2190500137@iau.edu.sa (A.K.); tsabualait@iau.edu.sa (T.S.A.); qmuaidi@iau.edu.sa (Q.M.); 2 Department of Pharmacy Practice, College of Clinical Pharmacy, Imam Abdulrahman Bin Faisal University, Dammam 31451, Saudi Arabia; rjchacko@iau.edu.sa

**Keywords:** core muscles, swimming performance, tensiomyography, sports training

## Abstract

Background: This study aimed to investigate the efficacy of core training in the swimming performance and neuromuscular properties of young swimmers. Methods: Eighteen healthy male swimmers (age: 13 ± 2 years, height: 159.6 ± 14.5 cm, weight: 48.7 ± 12.4 kg) were recruited from the Public Authority for Sports swimming pool in Dammam and randomly assigned to the experimental and control groups. The experimental group performed a six-week core-training program consisting of seven exercises (three times/week) with regular swimming training. The control group maintained its regular training. Swimming performance and neuromuscular parameters were measured pre- and post-interventions. Results: The experimental group benefitted from the intervention in terms of the 50 m swim time (−1.4 s; 95% confidence interval −2.4 to −0.5) compared with the control group. The experimental group also showed improved swimming velocity (+0.1 m.s^−1^), stroke rate (−2.8 cycle.min^−1^), stroke length (+0.2 m.cycle^−1^), stroke index (+0.4 m^2^·s^−1^), total strokes (−2.9 strokes), and contraction time for erector spinae (ES; −1.5 ms), latissimus dorsi (LD; −7 ms), and external obliques (EO; −1.9 ms). Maximal displacement ES (DM-ES) (+3.3 mm), LD (0.5 mm), and EO (+2.2 mm) were compared with the baseline values for the experimental group, and TC-ES (5.8 ms), LD (3.7 ms), EO (2.5 ms), DM-ES (0.2 mm), LD (−4.1 mm), and EO (−1.0 mm) were compared with the baseline values for the control group. The intergroup comparison was statistically significant (*p* < 0.05; DM-ES *p* > 0.05). Conclusion: The results indicate that a six-week core-training program with regular swimming training improved the neuromuscular properties and the 50 m freestyle swim performance of the experimental group compared with the control group.

## 1. Introduction

The main goal of competitive swimming is to cover a certain distance in the least possible time. Performance in swimming depends on producing propelling forces and reducing resistance to movement in the water [1]. Maintaining streamlined balance and body position is crucial in enhancing the proficiency of swimmers’ performance, which depends on the strength of the core muscles [2]. Several studies recommended adding core strength training to be an integral part of swimming training to improve performance [2,3,4]. Exercises to train core muscles can be exceptionally beneficial for sprint swimmers, allowing the effective transmission of force between the trunk and the upper and lower extremities to propel the body through the water, which leads to increased athletic performance and improved functional skills [5]. 

The enhancement of force production resulting from core training is achieved by improving neural adaptation, leading to faster nervous system activation, improved synchronisation of motor units, increased neural recruitment patterns, and lowered neural inhibitory reflexes [3,4]. These elements can be particularly beneficial for sprint swimmers [3]. Moreover, they reduce training costs and effort and help coaches choose the most appropriate training program to assess which swimmers could improve their performance effectively. Furthermore, they can reduce the rate of injuries by improving the efficacy of core muscles [3]. A strong core enables athletes to perform more effectively and conduct swift body movements, enhancing force distribution throughout the body [6]. A weak core leads to energy leakage, resulting in less powerful kicks and a decreased overall amount of power produced [7].

Previous studies focused on the effects of land-based limb power and strength interventions, with inconsistent findings with regard to swimming [8]. Furthermore, there is limited evidence regarding applying core-training programs to swimmers in terms of swimming performance. Weston et al. [9] reported an improvement in front crawl swimming time and core muscle functions following a 12-week isolated core-training program among young swimmers. A recent study by Karpiński et al. [10] also reported an improvement in swimming performance following a six-week core-training program among national-level Polish swimmers. Patil et al. [2] also reported an improvement in sprint time following six weeks of a core-training program in competitive swimmers. At the same time, Martens et al. [11] did not show a direct relationship between improvement in core muscle strength and swimming performance. There is also a lack of accurate performance measurements in core muscle training among swimmers [9,12]. Even though few studies are available that have assessed swimming speed, most of these studies did not assess accurate swimming parameters such as stroke rate, stroke length, sprint time, stroke index, etc., following the core-training program. It is also important to assess the performance parameters of the key core muscles that maintain the body position during swimming, such as the external oblique, erector spinae, and latissimus dorsi. 

Tensiomyography (TMG) is a novel and non-invasive neuromuscular measurement used to quantify the contractile properties of the muscles and provide information about how the muscles respond to the exercises and the load [13,14]. It has also been used to assess muscle stiffness and composition [15]. The assessment of the contractile properties of the muscles can provide valuable information about the adaptations of the muscles in response to the training. Several researchers used TMG to measure the effect of various training loads in soccer [16,17,18], volleyball [19,20], and basketball players [21] and monitor the effects of physical training in soccer and basketball players throughout the season [21,22]. TMG has also been used to establish the relationship between neuromuscular parameters and sports performance indicators in cyclists [23] soccer [24] and rugby players [25]. To the best of the authors’ knowledge, TMG has never been used to quantify the effect of core training among swimmers. Therefore, this study aimed to investigate the efficacy of a six-week core-training program in the swimming performance and neuromuscular properties of young swimmers. We hypothesised that an additional core-training program, along with a regular swimming training program, would lead to positive changes in the performance of young swimmers. This is the first study to use TMG to measure the effect of core-training exercises on swimmers.

## 2. Materials and Methods

### 2.1. Participants

**Figure 1:**
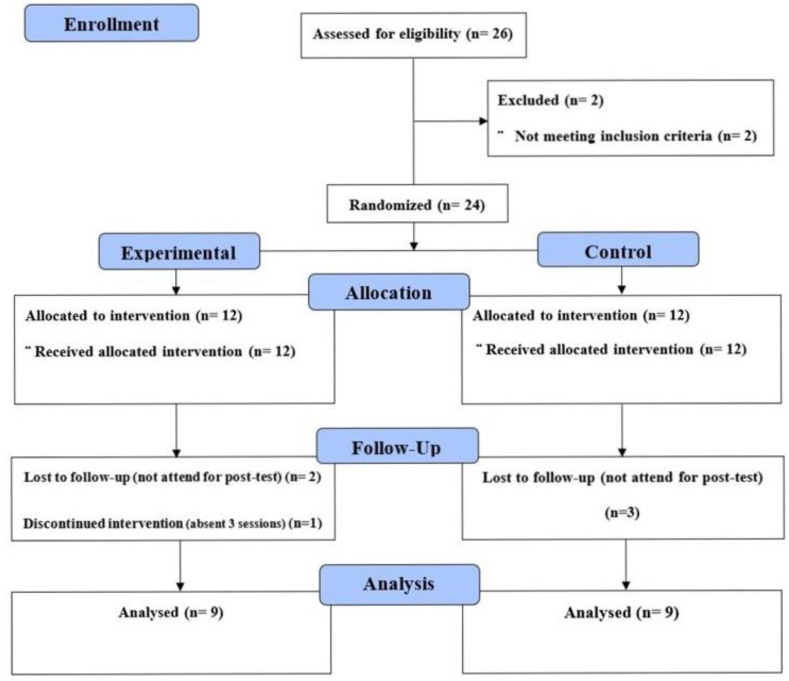
Consort diagram of participants’ flow.

Twenty-six healthy male young swimmers were approached for possible participation. Two subjects were excluded because they did not meet the inclusion criteria. Six subjects were dropped during the study because they did not attend the post-test and were absent from three training sessions (one participant). Nine male swimmers (age: 13 ± 2 years, height: 158.8 ± 17.3 cm, weight: 48.3 ± 14.2 kg, swimming experience 2.8 ± 0.4 years) were assigned to the experimental group and another nine male swimmers (age: 13.11 ± 2.6 years, height: 160.4 ± 11.9 cm, weight: 49.1 ± 11.3 kg, swimming experience 2.9 ± 0.7 years) to the control group (Figure 1). The inclusion criteria were as follows: (1) healthy subjects aged 10–16 years, (2) normal body mass index, and (3) swimming experience of more than one year. The exclusion criteria were as follows: (1) any previous injury of the shoulder or back muscles that could affect the training or measurement as reported by the participant, (2) any neurological or systemic disease as reported by the participant, (3) biomechanical abnormality of the participant that could affect the training and measurements, and (4) any medication that could affect performance. At baseline, the swimmers in both groups were performing a similar regular training program as they were enrolled under the same coach. The regular training consisted of seven sessions of pool-based training and two sessions of a land-based training program. The participants were covering an equal swimming distance per week with an average of 28 km. The major component of the regular training was endurance training. All the participants were familiar with core training, but not actively engaged in any core-training program. The idea of the research (including benefits, risks, and time needed) and the procedures were explained to the swimmers and their legal guardians, and written informed consent was taken from them. The participants were randomly assigned to two groups equally using the envelope drawing method, in which the envelope contained a number pertaining to the groups. Randomisation was conducted by a researcher who was unrelated to the study. Ethical approval was obtained from the Institutional Review Board of Imam Abdulrahman Bin Faisal University (IRB-PGS-2019-03-241).

### 2.2. Design

This study followed a randomised control trial with a pre-test–post-test control group (parallel design). The experiment was performed in the swimming pool of the Public Authority for Sports in Dammam. The calculation of the sample size was based on a previous randomised control trial by S. Girold et al. [26], who examined the effect of speed training exercises on a 50 m swim time (ST) by calculating the mean and standard deviation (SD) of the two groups, with 80% power and 0.05 type 1 error. Group 1 (experimental *n* = 11) had a mean of 29.69 and SD of (2.45), and Group 2 (control *n* = 11) had a mean of 32.85 and SD of (2.77). The ratio between the two groups was 1:1, with a 0.05 significance level (alpha) and 80% power. The results showed that 18 subjects were required, with nine subjects in each group.

### 2.3. Procedure

The experimental group performed a six-week core-training program in addition to regular training, and the control group continued their regular training. The participants were asked not to participate in any research or training other than their regular swimming training during the study. The swimmer’s dominant side was chosen for measurement. The dominant side is the same as the hand used in writing. TMG and swimming parameter measurements for the control group were performed twice: before the research began and after six weeks. For the experimental group, these were performed before and after the intervention. The reporting of this study followed the Consolidated Standards of Reporting Trials guidelines [27].

### 2.4. Intervention

The exercise program was based on existing literature and continued for 18 sessions for six weeks (three sessions per week, one hour per session, lasting no more than one hour in total) [9,28]. The progression of the exercise was performed by gradually increasing the number of repetitions and sets and the level of resistance/hold time (Table 1). All exercises were performed under the researchers’ supervision. All standard safety procedures were followed, including warm-up and cool-down exercises, rest time between exercises, and the use of correct techniques. The participants had to perform 10 min of warm-up exercises (i.e., bent over twist, criss-cross crunches, forward shoulder rotation, and backward shoulder rotation) followed by 30 min of seven core exercises. A 1 min rest was given between the exercises and a 10 min rest after the core exercises. One hour of regular training then followed. Finally, the swimmers performed cool-down exercises. Table 1 presents the details of the training program.

### 2.5. Outcome Measures

#### 2.5.1. Neuromuscular Properties

TMG was used to determine the neuromuscular properties of the external oblique (EO), erector spinae (ES), and latissimus dorsi (LD) muscles. These muscles were chosen for the measurement because they are the key muscles to maintain position during swimming and affect core stability and strength [9]. Moreover, our training program targeted these muscles. The LD is an extrinsic back muscle that contributes to arm adduction, extension movement, and stabilising the trunk during movement. The EO contributes to stabilising trunk movement and maintaining abdominal pressure and anatomical position. The ES muscle acts as a stabiliser for the entire vertebral column and the cranio-cervical region [29]. The participants were asked to remain in a relaxed supine position during measurement of the EO muscle, whereas a relaxed prone position was used to measure the ES and LD muscles [30]. The TMG sensor was positioned with regard to the muscle being measured, with a spring constant of 0.17 N mm^−1^. Two self-adhesive stimulating electrodes were positioned at proximal and distal distances of 1.5 cm from the sensor. An initial stimulation current of 10 mA was used with a single square wave monophasic 1 ms-long pulse, and the stimulation was gradually increased by 10 mA until no more increase in the response amplitude or when the maximal stimulator output was reached. To prevent the effects of muscular fatigue and potentiation, an interval of ≥10 s was preferred between consecutive measurements. TMG software instantaneously showed muscle displacement in the form of a graph and recorded it. The total test time was 30 min. The validity and reliability of TMG has been established by measuring neuromuscular properties [28]. TMG has five outcome measures: TC, TS, TD, DM, and ½ TR. In this study, we used TC and DM as the key parameters because they have high reliability in representing the effect of training on muscles. Contraction time is the period between the moment when muscular contraction is 10% and the moment when contraction reaches 90% of the maximum (ms), with the value of the TC depending on the percentage of slow or fast muscle fibres. The amplitude of DM (mm) is also linked to the TC values and depends on muscular flexibility [16].

#### 2.5.2. Swimming Parameters

**Figure 2:**
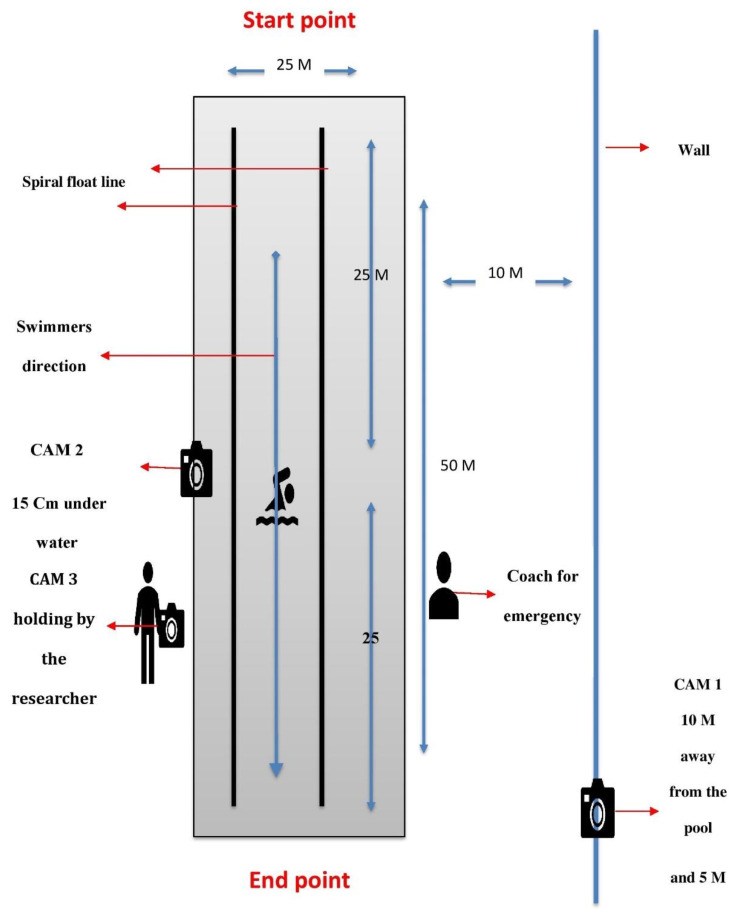
Swimming performance measurements.

All swimming performance measurements were taken in the same indoor swimming pool (50 m) at a temperature of 30 °C. Safety procedures were followed to ensure minimal risk, and this included a rescue team being available in the swimming pool. Furthermore, safety equipment, such as safety ropes and safety rings, was available in the swimming pool to prevent the risk of drowning. Special preparations were followed to determine the stroke parameters, including setting up a straight-line sector in the swimming pool [31]. Divider markers in the form of spiral float line lane ropes of 50 m were placed in the swimming pool. Three high-speed waterproof video cameras (GoPro HERO7, GoPro Inc, San Mateo, CA, USA) were used. One camera was placed on a wall 10 m away from the swimming pool to detect the total ST from the start point to the end point. The second camera was placed 0.15 m underwater in the centre of the lane to detect the stroke parameters [31]. The third camera was movable by the researcher to keep track of the entire trial (Figure 2). A chronometer was used by the coach to detect the ST to avoid possible time error. This measure is widely used to identify the stroke parameters [31,32]. To calculate the stroke parameters, the swimmers were asked to perform 15 min of warm-up exercises before the trial. Each swimmer was asked to take up a starting position in the swimming pool, followed by a 50 m front crawl swim with a maximum performance from a push-off start from the wall at the surface level (to isolate the effect of a dive) to the 50 m end point. The swimmers were asked to swim in a straight line at the whistle signal. Data were saved on the investigator’s laptop with password protection, which means that only the researchers had access to the information. The trial measurement time took a total of 1.5 h. After taking the measurements, we used Pinnacle 22 software (https://www.pinnaclesys.com, accessed on 23 January 2021) to analyse the stroke parameters by viewing the trials and calculating the strokes. This program is valid and reliable [33].

The following parameters were measured:

1. Swimming velocity or swimming speed (SV; m.s^−1^): Total distance covered (50 m) divided by the time required to cover that distance (s) [31,34,35] $\left(\mathrm{SV}=\frac{D}{T}\right)$;
2. Stroke rate (SR; cycle.min^−1^): The average time to perform a complete arm stroke *SR* = (T _stroke_^−1^) × 60. SR was measured by taking the average of three complete cycles divided by three, and we used the equal sign in the equation [31,34,35]. SR = (3/3 CYCLE TIME) × 60;
3. Stroke length (SL; m. cycle^−1^): SV (m.s^−1^) divided by SR (cycle.min^−1^) yields the cycle length in meters [31,34,35]. $SL=\left(\frac{V}{SR}\right)$ *× 60;*
4. Total strokes (NX): The total of the arm stroke cycles performed during the maximal test divided by the total test time [31,34,35]. N = (SR × T) / 60;
5. Sprint time (ST)*:* The total time (s) covered in a 50 m distance from start to end [9];
6. Stroke index (SI; m^2^·s^−1^): SL (in m) and SV [36]. SI=(SL×V).

### 2.6. Program Compliance

Program compliance was assessed according to the swimmers’ rate of participation using an attendance log. We considered compliance in training and decided to exclude subjects who would miss 15% of the required exercise sessions (three sessions). The researchers and team coaches monitored the exercise program in both the experimental and control groups to encourage the swimmers and prevent the risk of any injury.

### 2.7. Statistical Analysis

Data are presented as the mean ± SD. The difference was obtained between the post-test and pre-test to quantify the changes in athletic performance with an estimated 95% confidence interval (CI). SPSS version 20.0 was used to analyse the effect of the core-training intervention on all the outcome measures. Analysis of covariance was used to compare the two groups, with body mass, age, and pre-test score as the covariates to manage any imbalances in the measures between the intervention and the control group at baseline [9,37]. The change in the baseline method was used to represent the absolute change in the mean within-group [38]. Probabilistic magnitude-based inferences were made about the true value of the outcomes based on the likelihood that the true population difference was considerably positive or substantially negative [9,39]. The magnitude thresholds that were used as thresholds were 0.1, 0.3, and 0.5 for small, moderate, and large correlation coefficients, respectively, as suggested by Cohen [40] (1988). The standardised thresholds of 0.20, 0.60, and 1.20 [9,37] for the standardised differences in the means (the mean difference divided by the between-subjects SD) were derived from the between-subjects standard deviations of the baseline value to assess the magnitude of the effects. Effect size = mean difference (X_1_ – X_2_)/pooled SD. Statistical analysis was set to a significance level of *p* ≤ 0.05. The normal distribution for the data was tested using the Shapiro–Wilks test, observation of histograms, and Q-Q plots.

## 3. Results

Table 2 presents the characteristics of the subjects. No statistically significant difference was found between the groups. Table 3 shows the core-training intervention, which had a positive effect on the swimming performance outcomes and neuromuscular outcomes. TC-ES, TC-LD, and SL showed a large beneficial effect after the training with the effect size *d* = 1.47, 1.01, and 0.7, respectively. TC-EO (effect size *d* = 0.5), DM-ES (effect size *d* = 0.33), DM-LD (effect size *d* = 0.45), DM-OE (effect size *d* = 0.5), SI (effect size *d* = 0.5), and NX (effect size *d* = 0.4) showed a moderate beneficial effect, while SR (effect size *d* = 0.3) and SV (effect size *d* = 0.28) showed a small effect. The group comparison was statistically significant (*p* ≤ 0.01) in all the swimming performance outcomes. Moreover, the core-training intervention had a beneficial effect on ES, LD, and EO, with TC reduced and DM increased. The control group showed an increase in TC-ES, TC-LD, TC-EO, and DM-ES and a decrease in DM-LD and DM-EO. The group comparison after the core training was statistically significant (*p* ≤ 0.05) for TC-ES, TC-LD, TC-EO, DM-LD, and DM-EO.

## 4. Discussion

This study investigated the efficacy of a six-week core-training program in the swimming performance and TMG neuromuscular properties of young swimmers. To the best of our knowledge, previous studies have focused mostly on the effects of land-based limb power and strength interventions, with inconsistent findings with regard to swimming [8]. Moreover, there is limited evidence regarding the application of a core-training program to swimmers in terms of functional performance. Moreover, no study has used TMG to measure the effect of core-training exercises on swimmers. In our study, the core-training program had a beneficial effect on swimming performance and neuromuscular outcomes compared with the control group. Therefore, our finding rejected the null hypothesis and agreed with the alternate hypothesis that a six-week core-training program significantly affects the swimming performance parameters and TMG neuromuscular parameters of young swimmers (*p* < 0.05).

### 4.1. Swimming Performance and Core Training

The change in the swimming performance time required to improve swimmers’ performance was 0.5% [9,39]. The beneficial effect was (−1.4 s) ≅ 3.47% on sprint swimming performance time after the core-training program, as calculated by $\frac{\mathrm{post}-\mathrm{pre}}{\mathrm{pre}}\times 100$, which represents the estimated percentage differences [9,39]. Furthermore, SV (+0.1 m.s^−1^), SR (−2.8 cycle.min^−1^), SL (+0.2 m. cycle^−1^), SI (+0.4 m^2^·s^−1^), and NX (−2.9 total strokes) represented a positive performance improvement [41]. These results showed that the beneficial effect was moderate on SL, SI, and NX and small on ST, SR, and SV after the training. The group comparison (95%) CI was statistically significant (*p* ≤ 0.01) for ST, SV, SR, SL, SI, and NX. These findings are consistent with those of previous studies [2,9,32].

Weston et al. [9] studied the effect of 12 weeks of core training on swimming performance, which included ST and the physiological change in core muscles assessed by electromyography. The findings showed a significant improvement in ST and core muscle function starting from the sixth week. Moreover, the beneficial effect of ST was positively large. In the present study, the effect was positively small. Nevertheless, the variation in effect size depended on the mean difference between the groups and the mean difference method. Additionally, it was not associated with the p-value, as it could be beneficial, but not significant [42]. D. Patil et al. [2] reported that a six-week core-training program could significantly enhance swimming performance, including ST, SV, and SI, among young competitive swimmers in the 50 m freestyle. S. Iizuka et al. [32] reported that trunk muscle training could significantly improve swimming performance by reducing the starting time. The current study results are not consistent with those of H. Tanaka et al. [43], who reported that land-based training did not improve swimming performance and that improved power and strength did not affect swimming performance. This variation in the results may be due to the methodological issues in Tanaka et al.’s study, as they did not use a control group to measure the effect of the intervention on the group. A possible explanation for our result is that core training can enhance the production and transmutation of forces in the lower and upper extremities [3,4,5]. The enhancement of force production in core training is achieved by improving neural adaptation, leading to faster nervous system activation, improved synchronisation of motor units, increased neural recruitment patterns, and decreased neural inhibitory reflexes [3]. These can help sustain the efficient posture needed while swimming, thus generating powerful movement effectively. They can also help to stabilise the pelvis and the lumbar spine during kicks, enabling powerful strokes and assisting swimmers to move faster in water. They can also help the abdominal muscles maintain balance in the water and stabilise the trunk to prevent sagging down, which leads to decreased drag force [2]. If these aspects are ignored, resistive forces at the extremities increase, and the stroke technique breaks down, leading to inefficient strokes [44]. In our study, the core power of the swimmers increased, which enhanced their capability to sustain an efficient technique throughout the entire race.

### 4.2. Swimming Parameters

The swimming parameters such as ST, SV, SR, SL, SI, and NX are connected. For example, SV and SI increase when SL increases and SR decreases; NX increases SV; SI increases when SL increases, with no change in SR, in freestyle swimmers [45]. SR, SL, and SV are the main swimming parameters in the 50 m freestyle that demonstrate improvement in swimming performance [33,46]. As previously mentioned, our results showed that the SR value for the experimental group decreased by 3.5 and that SL, SV, and SI increased by 0.2 m. cycle^−1^, 0.06 m.s^−1^, and 0.4 m^2^·s^−1^, respectively. The control group showed a decrease of 0.7 cycle.min^−1^ in SR and an increase of 0.02 m. cycle^−1^, 0.004 m.s^−1^, and 0.03 m^2^·s^−1^ in SL, SV, and SI, respectively. The group comparison was SR (−2.8) *p* < 0.01; SL (0.2) *p* < 0.00; SV (0.1) *p* < 0.00; and SI (0.4) *p* < 0.00. Our findings are similar to those in previous studies [43,45,46,47,48].

Hay et al. [47] showed that an increase in SL was associated with improved SV, which led to performance enhancement. Thus, a decrease in SL is linked to a decrease in SV [45]. Girold et al. [48], Aspenes et al. [49], and Roberts et al. [50] also found that the improvement in performance after training was associated with increased SL and decreased SR. The results of the current study are not consistent with those of some previous studies [26,43]. Tanaka et al. [43] found that an increase in performance did not result in an increase in SL. Girold et al. [26] found a significant decrease in SL with a significant increase in SR. This variation in results may be due to the training program, as previous studies used static sprint training and resistance training focused on the arms and shoulders. The authors suggested that low repetitions with a high SV are needed for improving SL. Increasing the strength level could lead to an increased SL [46]. Furthermore, as swimming distance increases to >400 m, SR becomes less of a determining factor [51], but increases because of a decrease in SL at volitional exhaustion [45]. Moreover, SL tends to be shorter for butterfly and longer for backstroke and freestyle, while SR is less in backstroke and freestyle and increases in butterfly and breaststroke. These may depend on neuromotor control and muscular endurance [44]. Our findings support the idea that an increase in SL and a decrease in SR represent an improvement in ST and swimmers’ speed, leading to improvement in SI.

### 4.3. Neuromuscular Properties

Our intervention improved core muscle functionality, which was assessed by the TMG parameters. TC decreased (−1.5 ms) for ES, (−7 ms) LD, and (−1.9 ms) EO, and DM increased (+3.3 mm) for ES, (+0.5 mm) LD, and (+2.2 mm) EO compared with the baseline value. The control group was (5.8 ms) TC-ES, (3.7 ms) LD, (2.5 ms) EO, (0.2 mm) DM-ES, (−4.1 mm) LD, and (−1.0 mm) EO compared with the baseline value. The beneficial effect was large for TC-ES and TC-LD and moderate for TC-EO, DM-ES, DM-LD, and DM-EO. The group comparison (95% CI) was statistically significant *p* ≤ 0.05 for TC-ES, TC-LD, TC-EO, DM-LD, and DM-EO and *p* ≤ 0.12 for DM-ES. We assumed that the increase in TC and the decrease in DM for the control group were due to the slow process of recruiting motor units [16]. Our findings are consistent with some previous studies [16,22,52].

Rusu et al. [16] showed that a six-week isometric–concentric training could improve muscle functionality associated with a decrease in TC and an increase in DM values, leading to a high rate of Type II muscle fibres and a faster process of motor unit recruiting for the experimental group. Conversely, the control group showed an increase in TC and a decrease in DM values, indicating a slow motor unit recruiting process. Valverde et al. [22] found that a decrease in TC and increased DM values indicated a good response following muscle training. Monteiro and Massuca [52] showed that a decrease in muscle TC and an increase in DM had a beneficial effect on the muscle following training. Rusu et al. [16] suggested that concentric training could lead to an increased TC and a reduced DM, which is the opposite effect of isometric–concentric training and could occur due to the enlargement of the muscle as a result of concentric training. Muscle fatigue could change the TMG parameters, as it could increase TC and decrease DM as a result of high-intensity resistance, interval training, or endurance training over short periods [53]. However, our findings support that a decrease in TC and an increase in DM following isometric–concentric training could improve muscle functionality.

### 4.4. Practical Application

The results showed that a core-training intervention can enhance swimmers’ performance and improve muscular functionality. The findings from this study can be generalised to any swimmer in the age group studied. Therefore, our findings can be helpful as evidence to assist coaches, trainers, and therapists in applying a core-training program along with regular training to improve the swimming performance of young swimmers. Nevertheless, this study has several limitations. First, puberty, maturation, and testosterone hormones could have influenced the results. They vary from child to child, cannot be controlled, and may mask training effects [54]. Further research should include or control for the effect of growth and maturation, which is a major hurdle when studying young athletes. Second, only male swimmers were recruited for the study, and thus, our findings cannot be generalised to female swimmers. Third, we investigated only the effect of a core-training intervention on the 50 m freestyle. We recommend that other swimming styles and long-distance swimming be included. Further studies are needed to investigate larger groups and athletes at different levels and those from different places or clubs. Fourth, some of the exercises used in the core-training program (e.g., shoulder press) might have caused an improvement in the upper and lower limb strength, which may influence the swimming performance. Fifth, we were unable to demonstrate the effect of the core-training intervention on stroke depth. Further research is needed to demonstrate the effect of core training on stroke depth. Nevertheless, we found a significant improvement in our performance measures after considering the effects of body mass and age.

## 5. Conclusions

A six-week core-training program along with regular swimming training significantly improved the freestyle swimming performance and core muscle properties, such as contractility (contraction time), excitability, extensibility, and elasticity, of the experimental group compared with the group that did not undergo the core-training program. These results can serve as evidence to assist coaches, trainers, and therapists in improving the swimming performance of young swimmers.

## Figures and Tables

**Table 1:** Core training program for the swimmers.

		Week 1			Week 2			Week 3		
Exercises	Progression	Repetitions	Sets	Weight	Repetitions	Sets	Weight	Repetitions	Sets	Weight
Prone Bridge *	Volume	30 s Hold	Two	-	60 s Hold	Two	-	90 s Hold	Two	-
Side Bridge *	Volume	30 s Hold	Two	-	60 s Hold	Two	-	90 s Hold	Two	-
Bird Dog *	Volume	10 Repetitions	Three	-	15 Repetitions	Three	-	20 Repetitions	Three	-
Leg Raising *	Volume	10 Repetitions	Three	-	15 Repetitions	Three	-	20 Repetitions	Three	-
Overhead Squat **	Resistance	10 Repetitions	Three	3 kg	10 Repetitions	Three	4 kg	15 Repetitions	Three	5 kg
Sit Twist **	Resistance	15 Repetitions	Three	3 kg	15 Repetitions	Three	4 kg	15 Repetitions	Three	5 kg
Shoulder Press ***	Volume	10 Repetitions	Three	3 kg	10 Repetitions	Four	3 kg	15 Repetitions	Three	3 kg
		Week Four			Week Five			Week Six		
Prone Bridge *	Volume	90 s Hold	Three	-	120 s Hold	Two	-	120 s Hold	Three	-
Side Bridge *	Volume	90 s Hold	Three	-	120 s Hold	Two	-	120 s Hold	Three	-
Bird Dog *	Volume	25 Repetitions	Three	-	25 Repetitions	Four	-	30 Repetitions	Three	-
Leg Raising *	Volume	25 Repetitions	Three	-	25 Repetitions	Four	-	30 Repetitions	Three	-
Overhead Squat **	Resistance	20 Repetitions	Three	6 kg	20 Repetitions	Four	7 kg	25 Repetitions	Three	7 kg
Sit Twist **	Resistance	20 Repetitions	Three	6 kg	20 Repetitions	Four	7 kg	25 Repetitions	Three	7 kg
Shoulder Press ***	Volume	20 Repetitions	Three	3 kg	20 Repetitions	Four	3 kg	25 Repetitions	Three	3 kg

* The exercises were performed by body weight. ** The exercises were performed with a medicine ball. *** The exercises were performed with a dumbbell. Core-training exercise details: Prone bridge (plank): hold a straight body position and use elbow and toes to support and pull abdomen and back in a natural position. Side bridge (side plank): start by lying on one side and push the body line straight through the feet and hips. Bird dog: stand on one knee and opposite hand, back in normal position, slowly extend one leg backwards, and raise opposite arm horizontally to the back level. The pelvis should not tilt and back arm should go to start position, and repeat on the other side. Leg raise: Lie supine on the floor with extended knees and lift one leg straight till 75-degree hip, then return to start position and swap the other side. Overhead squat: stand on both legs and hold medicine ball overhead using both hands; back should be vertical and straight. Squat as low as possible while maintaining balance. Sit twist: sit on the floor with knees bent 45 degrees and keep in natural position. While holding a medicine ball with both hands, twist the waist and shoulder to one side. The ball should be held out and front. Return to the neutral position, and repeat the other side. Shoulder press: lie prone with extended arms, hold a dumbbell in both hands, raise one arm, return, and repeat the other arm.

**Table 2:** Characteristics of the subjects. No statistically significant difference was found between the group. Values are presented as mean ± standard deviation.

Variable	Core Training Group (*n* = 9)	Control Group (*n* = 9)
Age (year)	13 ± 2	13.11 ± 2.6
Body Weight (kg)	48.3 ± 14.2	49.1 ± 11.3
Height (cm)	158.8 ± 17.3	160.4 ± 11.9

**Table 3:** The outcome measures between- and within-group comparison.

	Core Training Group			Control Group			Group Comparison		
**Performance Parameters**	**Baseline Value**	**Post-Test Value**	**Adjusted Changed Score** **Lower—Upper 95% CI**	**Baseline Value**	**Post-Test Value**	**Adjusted Changed Score** **Lower—Upper 95% CI**	**Differences between Groups 95% CI**	**QI**	***p***
TC ES (ms)	17.4 ± 3.4	15.8 ± 2.7	−1.5; −6.5 to 3.4	20.8 ± 4.3	26.5 ± 9.7	5.8; 0.92 to 10.8	−7.3; −14.7 to −0.007	Large +	0.05
TC LD (ms)	26.7 ± 6.8	24.4 ± 4.7	−7; −11.3 to −2.7	39.7 ± 9.6	38.6 ± 5.7	3.7;−0.6 to 8	−10.7; −17.6 to −3.8	Large +	0.01
TC OE (ms)	22.3 ± 5.8	20.8 ± 5.8	−1.9; −5 to 1.1	24 ± 6.8	26.1 ± 3.1	2.5; −0.5 to 5.6	−4.5; −8.9 to −0.1	Moderate +	0.05
DM ES (mm)	14.3 ± 7.3	17.3 ± 8.8	3.3; 0.4 to 6.2	13.4 ± 7.6	13.8 ± 6.4	0.2; −2.7 to 3.1	3.1; −1 to 7.3	Moderate +	0.12
DM LD (mm)	14.8 ± 8.7	15.6 ± 9.5	0.5; −2.7 to 3.8	17.4 ± 7.6	13 ± 4.1	−4.1; −7.4 to −0.9	4.7; 0.01 to 9.3	Moderate +	0.05
DM OE (mm)	9.3 ± 6.7	11.6 ± 8.5	2.2; 0.1 to 4.4	7.7 ± 2.5	6.5 ± 2.3	−1.0; −3.2 to 1.1	3.3; 0.3 to 6.3	Moderate +	0.04
ST (seconds)	39.2 ± 8.4	37.6 ± 8.3	−1.6; −2.2 to −0.9	37.6 ± 4.0	37.5 ± 4.1	−0.1; −0.8 to 0.5	−1.4; −2.4 to −0.5	small +	0.01
SV (m.s^−1^)	1.3 + 0.3	1.3 ± 0.2	0.06; 0.03 to 0.1	1.3 ± 0.2	1.3 ± 0.1	0.004; −0.02 to 0.03	0.1; 0.02 to 0.1	Small +	<0.01
SR (cycle.min^−1^)	49.6 ± 8.0	46 ± 8.1	−3.5; −4.8 to −2.2	46.1 ± 4.3	45.4 ± 4.3	−0.7; −2 to 0.6	−2.8; −4.7 to −1	Small +	0.01
SL (m. cycle^−1^)	1.6 ± 0.3	2.5 ± 0.8	0.2; 0.2 to 0.3	1.8 ± 0.2	2.4 ± 0.5	0.02; −0.04 to 0.1	0.2; 0.1 to 0.3	Large +	<0.01
SI (m^2^·s^−1^)	2.2 + 0.7	1.8 ± 0.3	0.4; 0.3 to 0.5	2.4 + 0.5	1.8 ± 0.2	0.03; −0.1 to 0.2	0.4; 0.2 to 0.5	Moderate +	<0.01
NX	31.7 + 5.5	28.3 ± 5.5	−3.4; −4.2 to −2.6	28.8 + 4.3	28.4 ± 4.2	−0.5; −1.4 to 0.3	−2.9; −4.1 to −1.6	Moderate +	<0.01

QI, qualitative inference; CI, confidence interval; +, positive effect on core-training group compared with controls; TC, time contraction; DM, muscle displacement; ES, erector spinae; LD, latissimus dorsi; OE, obliques external; ST, sprint time; SV, swimming velocity; SL, stroke length; SR, stroke rate; SI, stroke index; NX, total strokes.

## Data Availability

The data that support the findings of this study are available from the corresponding author upon request.
